# Complement C3 in panvascular disease: a central integrator of immune signaling and vascular remodeling

**DOI:** 10.1042/CS20257865

**Published:** 2025-11-04

**Authors:** Yu Li, Hesong Zeng, Xiaodan Zhong

**Affiliations:** 1 Department of Cardiology, Tongji Hospital, Tongji Medical College, Huazhong University of Science and Technology, Wuhan, Hubei, 430030, China; 2 Hubei Provincial Engineering Research Center of Vascular Interventional Therapy, Wuhan, Hubei, 430030, China

**Keywords:** complement C3, immune–vascular interaction, panvascular disease, vascular remodeling, targeted therapy

## Abstract

Panvascular disease, defined by the systemic involvement of multiple vascular beds, poses a growing challenge to contemporary diagnostic and therapeutic paradigms. Despite organ-specific manifestations, these conditions share a convergent pathological basis driven by chronic low-grade inflammation, immune dysregulation, and maladaptive vascular remodeling. Within this immunovascular interface, complement C3 (C3) has emerged as a pivotal regulator. Positioned at the convergence of the classical, lectin, and alternative complement pathways, C3 integrates systemic immune cues with microenvironmental stimuli to orchestrate endothelial activation, smooth muscle cell phenotypic switching, immune cell recruitment, platelet activation, and fibroinflammatory remodeling. This review provides a comprehensive analysis of C3 biology, including its structural domains, activation cascades, and downstream effector functions. We examine the role of C3 across major vascular cell types, endothelial cells, vascular smooth muscle cells, innate and adaptive immune cells, platelets, and fibroblasts, highlighting how C3 signaling dynamically shapes both acute injury responses and chronic vascular adaptation. In disease-specific contexts, we delineate how C3 contributes to the pathogenesis of atherosclerosis, coronary artery disease, aortic aneurysm and dissection, hypertension, pulmonary arterial hypertension, peripheral vascular disease, stroke, and autoimmune- associated vasculitides. Special emphasis is placed on the dual-phase roles of C3, such as its injuryexacerbating effects in the acute phase of stroke versus its reparative functions in neuroregeneration. Finally, we review emerging therapeutic strategies targeting C3, with a focus on compstatin-based inhibitors, their pharmacological profiles, clinical trial progress, and immunological safety considerations. Collectively, this review reframes C3 as a master orchestrator of panvascular pathology and a promising target for precision immunomodulation across vascular systems.

## Introduction

Panvascular diseases represent a significant and emerging global health concern, characterized by systemic atherosclerotic involvement of multiple vascular beds, including the coronary, cerebral, renal, and peripheral arteries [1,2]. More broadly, the spectrum of panvascular diseases extends beyond atherosclerosis to encompass a wide range of conditions, including disorders of small vessels, microvasculature, and veins, as well as vascular complications associated with immune-mediated disorders [3]. Despite considerable advances in the management of isolated vascular diseases, panvascular diseases continue to pose major diagnostic and therapeutic challenges. Epidemiological studies, such as the REACH Registry, have underscored the high prevalence of multi-bed vascular involvement, emphasizing the need for integrated, system-wide therapeutic strategies [4–8]. The emergence of panvascular medicine responds to this need by introducing a holistic medical paradigm that transcends traditional organ-based approaches, advocating for integrated diagnosis and treatment strategies to more effectively manage complex, systemic vascular pathologies.

In recent years, advancing research on panvascular diseases has increasingly highlighted that chronic low-grade inflammation and persistent immune dysregulation are not merely concomitant phenomena but central driving forces in their pathogenesis [9]. Notably, vascular injury across different anatomical territories often exhibits a co-ordinated pattern of progression, suggesting a shared pathological foundation rooted in systemic immune abnormalities [10]. Immune mechanisms are intricately involved at multiple levels, including sustained release of pro-inflammatory mediators, endothelial dysfunction, and the dedifferentiation and proliferation of vascular smooth muscle cells (VSMCs), underscoring the pivotal role of immune signaling in the initiation and propagation of multisite vascular injury [11,12]. Within this context, the complement system, an integral part of innate immunity, has garnered increasing attention. Among complement components, complement C3 (C3) occupies a central role, functioning as a convergence point for the classical, lectin, and alternative pathways and mediating key pro-inflammatory and pro-thrombotic processes [13–15]. Dysregulated activation of complement C3 has been implicated in endothelial dysfunction, plaque instability, and tissue injury across various vascular territories, indicating its critical involvement in the systemic progression of panvascular diseases.

Although substantial progress has been made in elucidating the role of complement activation in localized vascular conditions, the contribution of complement C3 to systemic vascular cross-talk and multi-bed vascular pathology is only partially understood. This knowledge gap underscores the importance of further synthesis and clarification. Therefore, in this review, we systematically examine the emerging roles of complement C3 activation in panvascular diseases. First, we will briefly outline the structure, activation pathways, and biological functions of complement C3. Subsequently, we will discuss the specific mechanisms by which C3 activation contributes to vascular injury. Finally, we will address the therapeutic potential of targeting complement C3 in panvascular disease and highlight key research questions for future investigation.

## Activation of complement C3

Complement C3 is a structurally modular protein that acts as a pivotal node in the complement cascade. Its architecture consists of a β-chain forming a stable macroglobulin (MG) core and an α-chain containing regulatory domains such as the anaphylatoxin segment, CUB domain, thioester-containing domain (TED), and C345c domain [16,17]. This organization confers C3 with both conformational stability and the ability to undergo dynamic structural changes essential for immune function.

**Figure 1:**
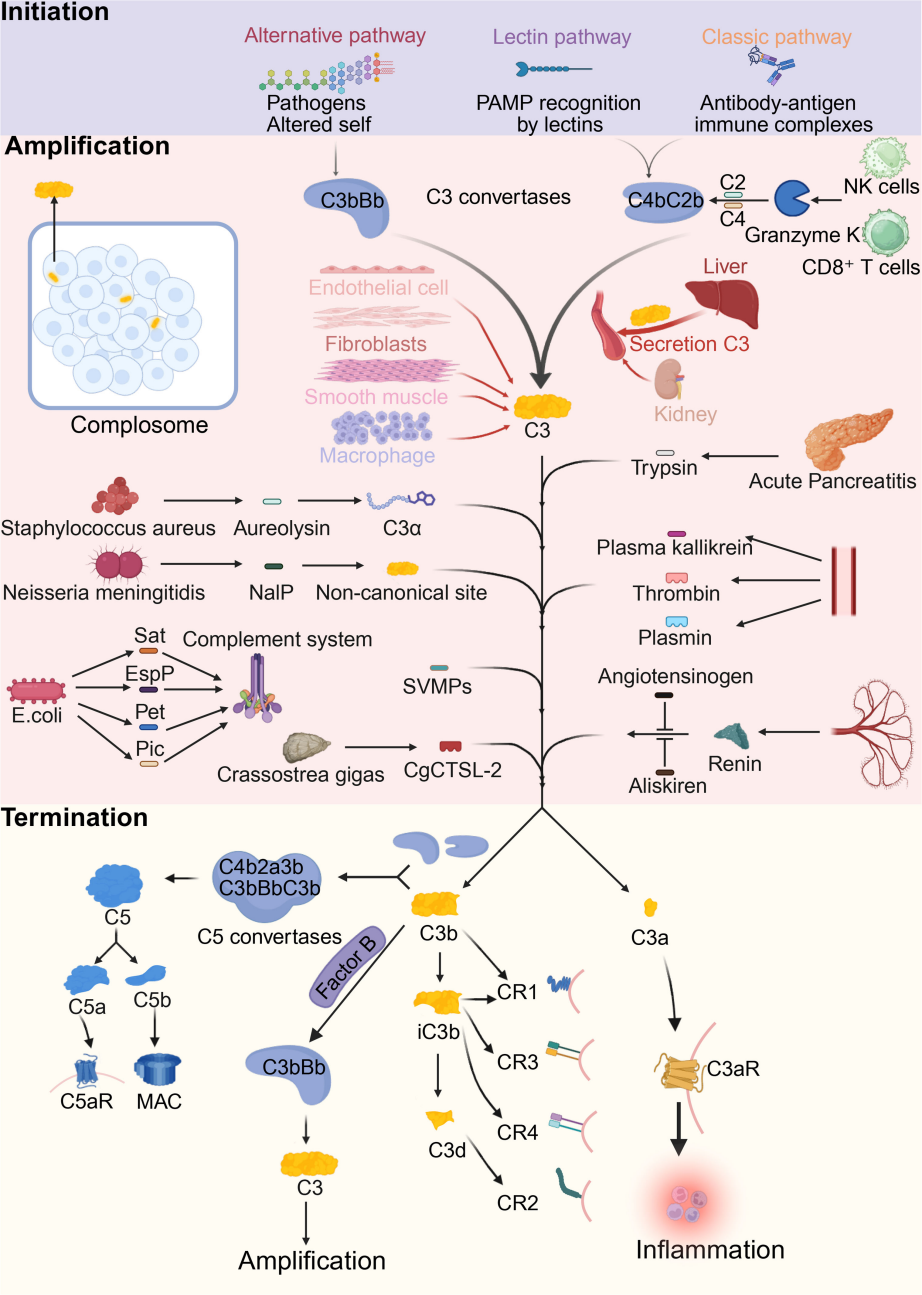
Canonical and non-canonical pathways of C3 activation and effector outcomes. Complement C3 integrates signals from three canonical pathways: the alternative pathway (triggered by pathogens or altered self), the lectin pathway (via lectin recognition of microbial patterns), and the classical pathway (via antibody-antigen immune complexes). C3 is also activated by intracellular C3 convertases (complosome) and by host- and pathogen-derived proteases, including granzyme K, trypsin, thrombin, plasmin, kallikrein, and microbial enzymes (e.g., aureolysin, NalP, CgCTSL-2, and SPATE family proteases). Locally produced C3 from non-hepatic cells further amplifies complement activity. Cleavage of C3 yields C3a, which promotes inflammation via C3aR, and C3b, which propagates amplification loops, contributes to C5 convertase assembly, and initiates formation of the membrane attack complex (MAC). C3b is further processed into iC3b, C3dg, and C3d, which modulate immune responses through complement receptors (CR1-CR4). C3, complement C3; C3a, complement C3a; C3b, complement C3b; C3bBb,alternative pathway C3 convertase; C4, complement C4; C2, complement C2; C4bC2b, classical/lectin pathway C3 convertase; C5, complement C5; C5a, complement C5a; C5b, complement C5b; C5aR, C5a receptor; CgCTSL-2, crassostrea gigas cathepsin L-like protease 2; CR1, complement receptor 1; CR2, complement receptor 2; CR3, complement receptor 3; CR4, complement receptor 4; C3aR, C3a receptor; EspP, extracellular serine protease, plasmid-encoded; iC3b, inactivated C3b; C3d, degradation fragment of C3; MAC, membrane attack complex; NaIP, Neisserial autotransporter lipoprotein protease; NK cells, natural killer cells; PAMP, pathogen-associated molecular patterns; Pet, Plasmid-encoded toxin; Pic, Protease involved in colonization; Sat, secreted autotransporter toxin; SVMPs, snake venom metalloproteinases.

Complement C3 is primarily synthesized by hepatocytes in the liver and secreted into the bloodstream as an inactive precursor under physiological conditions [18]. However, accumulating evidence indicates that C3 is also produced locally by a variety of extrahepatic tissues and cells, particularly under inflammatory or pathological conditions. Vascular endothelial cells, epithelial cells of barrier organs (such as the gut, lung, and kidney), fibroblasts, and immune cells including monocytes, macrophages, dendritic cells, and T lymphocytes have all been shown to express C3 in response to pro-inflammatory stimuli such as interleukin-1 beta (IL-1β), tumor necrosis factor-alpha (TNF-α), and Toll-like receptor ligands [19–22]. Local synthesis of C3 enables tissue-specific modulation of immune responses and contributes to amplification of inflammatory cascades independent of systemic complement levels (Figure 1).

Upon activation, complement C3 is proteolytically cleaved by C3 convertases (C4b2a from the classical/lectin pathways or C3bBb from the alternative pathway) into two principal fragments: C3a and C3b [23,24]. The small fragment C3a, released from the anaphylatoxin domain, acts as a potent pro-inflammatory mediator by binding to the G-protein-coupled receptor C3aR, thereby modulating immune cell activation, chemotaxis, and vascular permeability [18]. Meanwhile, the larger fragment C3b undergoes a substantial conformational rearrangement, exposing the reactive thioester group within the TED domain, which facilitates the covalent attachment of C3b to nearby pathogen surfaces, apoptotic cells, or damaged tissues [19].

Surface-bound C3b plays a pivotal role in amplifying the complement cascade. It associates with factor B, which, upon cleavage by factor D, forms the alternative-pathway C3 convertase (C3bBb), establishing a positive feedback loop that enhances further C3 activation [25]. In addition, the binding of an additional C3b molecule to the convertase forms the complement C5 (C5) convertase complex (C3bBb3b or C4b2a3b), which initiates terminal pathway activation and drives the assembly of the membrane attack complex (MAC) [26].

C3b activity is tightly regulated by complement control proteins, particularly factor I, which cleaves C3b into inactive yet opsonically active fragments such as iC3b, C3dg, and C3d [27]. Although these degradation products lose the ability to form convertases, they retain crucial roles in opsonization, immune complex clearance, co-stimulation of B cells, and the promotion of adaptive immune memory through engagement with complement receptors [14–17,27].

In addition to the well-established classical, lectin, and alternative complement activation cascades, recent advances over the past decade have delineated a non-canonical proteolytic axis whereby complement C3 is cleaved independently of traditional C3 convertases. This emergent ‘fourth pathway’ is mediated by a diverse array of host-derived, pathogen-encoded, and exogenous proteases, which collectively broaden the mechanistic scope of complement regulation and immunological cross-talk [28–34] (Table 1).

**Table 1:** Proteases involved in complement C3 cleavage and their immunobiological consequences

Proteases	Origin	Cleavage products	Biological consequences	Cleavage site(s)	References
Aureolysin	*Staphylococcus aureus*	C3a + SN and C3b2SN	Complement activation modulation, immune evasion.	C3α (Asn-Leu)	[32]
NalP	*Neisseria meningitidis*	C3a-like and C3b-like	Reduced C3b deposition, immune evasion.	C3α (Lys - Gly)	[33]
Sat, EspP, Pet, Pic	*Escherichia coli*	C3a, C3b, and iC3b	MAC formation inhibition, reduced C3b deposition, enhanced bacterial survival.	C3α (Arg - Ser), C3γ, C3α (non-canonical cleavage site)	[34]
Granzyme K	CD8 + T cells and NK cells	C4b, C2b, C3 convertase, C3b, C3a, and C5a	C4 and C2 cleavage, C3 convertase formation, Increased inflammation, Immune response modulation.	C3α (Arg - Ser)	[28]
Renin	Granular cells in the kidneys	C3a and C3b	Complement activation, immune enhancement, inflammation promotion, immune tolerance regulation.	C3α	[30,35,36]
Trypsin	Pancreatic cells of acute pancreatitis	C3a and C3b	Leukocyte activation, immune response modulation.	C3α (Arg - Ser)	[37]
Plasma kallikrein	Hepatocytes	C3a and C3b	Enhance inflammation, reduce immune evasion.	C3α (non-canonical cleavage site)	[29]
Thrombin	Prothrombin in plasma	C3a and C3b-like	Immune activation, regulation of the coagulation system	C3α (Arg - Ser)	[38]
Plasmin	Plasminogen in plasma	C3a and C3b	Regulation of immune response, promotion of inflammation.	C3α (Arg - Ser)	[38]
Micrurus spp. venoms	Micrurus venoms	C3a-like and C3b-like	Immune evasion, disruption of immune response.	C3α (non-canonical cleavage site)	[39]
CTSL	Lysosomal enzymes	C3a and C3b	Sustaining T cell survival, maintaining immune homeostasis	C3α	[40–42]

Host serine proteases such as kallikrein and granzyme K (GZMK), along with digestive enzymes like trypsin under pathological leakage, have demonstrated the capacity to cleave C3 at or near the classical convertase site, thereby generating bioactive C3a and C3b fragments [28,29,37]. Kallikrein cleaves C3 at the canonical Arg748-Ser749 bond, yielding functionally intact C3a/C3b and concomitantly activating the alternative pathway via factor B cleavage and C3bBb convertase assembly. In the setting of fungal infections, notably *Candida albicans*, kallikrein activation facilitates localized C3 deposition, enhancing fungal clearance through opsonophagocytic pathways [29]. GZMK, a cytotoxic granule protease secreted by memory-like CD8^+^ T cells and natural killer (NK) cells, has been shown to cleave C4 and C2, generating a functional C4b2b complex capable of mediating C3 activation independently of C1q or mannose-binding lectin (MBL) recognition [28]. This mechanism has been implicated in the pathogenesis of immune-mediated conditions such as rheumatoid arthritis and contact dermatitis, providing a direct mechanistic link between adaptive cytotoxic lymphocytes and innate complement activation [28,43]. Thrombin, coagulation factors XIa, Xa, IXa, and plasmin also cleave C3, acting as non-traditional C3 convertases that link the complement system to coagulation and fibrinolysis and promote inflammatory cell recruitment [38].

Pathogen-derived proteases also constitute potent modulators of the complement system. Exogenous proteases, including venom-derived snake venom metalloproteinases (SVMPs) from *Micrurus* species (e.g., *M. ibiboboca*), directly cleave C3, C4, and C5 in the absence of other complement components [39]. The resultant elevation of C3a and C5a, coupled with systemic complement consumption, implicates these enzymes in inflammation amplification and toxin dissemination [31]. In invertebrates, CgCTSL-2, a cysteine cathepsin protease from Crassostrea gigas, has been identified as a novel C3-cleaving enzyme mediating complement-dependent microbial clearance [44]. *Staphylococcus aureus* metalloprotease Aureolysin selectively cleaves the C3α-chain, transiently impairing opsonization despite reduced stability of the resulting C3b [32]. *Neisseria meningitidis* Neisseria autotransporter lipoprotein protease cleaves C3 at an atypical upstream site, yielding pseudo-C3a/C3b fragments with attenuated capacity for amplification, thereby promoting immune evasion in serum [33]. Among the SPATE family, proteases such as plasmid-encoded toxin (Pet), extracellular serine protease, plasmid-encoded (EspP), secreted autotransporter toxin (Sat), and protease involved in colonization (Pic) exhibit broad complement-degrading activity. Pet cleaves C3, C5, and C9, disrupting MAC assembly; EspP degrades C3b and C5, inhibiting chemotaxis and phagocytic responses; Sat exhibits pan-complementolytic activity targeting C1q through C9; Pic, through cofactor-enhanced cleavage of C3b, compromises opsonin integrity and facilitates epithelial adherence by enteroaggregative *Escherichia coli* (EAEC) [31,34].

Further expanding the landscape of non-canonical C3 activation, Karpman’s group reported that all renins (plasma renin, kidney renin, and recombinant renin) could directly cleave C3 at the canonical convertase site, producing biologically active C3a and C3b in an aliskiren-sensitive manner [35]. However, Zhang et al. challenged these findings, citing the absence of a correlation between plasma renin and C3 levels in patients with C3 glomerulopathy (C3G) or atypical hemolytic uremic syndrome (aHUS), and demonstrating that recombinant renin did not cleave C3 *in vitro* [30]. Moreover, aliskiren failed to alter complement dysregulation in patient sera, and molecular modeling suggested that C3 is not positioned favorably within the active site of renin for catalytic cleavage. They further suggested that prior results could be explained by trypsin contamination during protein preparation. In response, Karpman’s group presented additional evidence ruling out trypsin contamination, confirmed aliskiren-sensitive C3 cleavage under multiple conditions, and showed that angiotensinogen competitively inhibits this cleavage, indicating potential tissue-specific relevance in the kidney where local renin levels are high and angiotensinogen may be depleted [36].

Collectively, these findings highlight a previously underappreciated complexity in complement regulation, where C3 activation can be orchestrated by a diverse array of non-canonical proteases under specific pathophysiological conditions. This additional mode of complement activation reveals the dynamic, spatially restricted, and context-dependent nature of C3 regulation, with significant implications for immune homeostasis, host–pathogen interactions, and the development of panvascular and chronic inflammatory diseases.

Recent studies have revealed that the functions of the complement system extend far beyond its classical role in extracellular immune defense. Intracellular complement activity, especially that of C3, has been identified in various cell types and shown to play critical roles in regulating cellular metabolism, inflammatory responses, and survival [22,45]. This intracellular complement network, now referred to as the complosome, is reshaping our understanding of immune regulation. Beyond hepatic synthesis, T cells, dendritic cells, epithelial cells, and hematopoietic stem/progenitor cells can produce C3 locally, either retaining it intracellularly without secretion or acquiring it via endocytosis [46,47]. Within these cells, C3 can be cleaved by intracellular convertase activity involving lysosomal proteases such as cathepsin L, generating the active fragments C3a and C3b [46]. Intracellular C3a engages C3a receptors (C3aR) localized on lysosomal or mitochondrial membranes, initiating downstream signaling cascades. Similarly, C3b interacts with intracellular regulatory proteins such as CD46, modulating cellular proliferation and inflammatory pathways [46]. In T cells, lysosomal cleavage of C3 generates C3a, activating the mTOR signaling pathway to support cell survival [45]. In fibroblasts, the C3a–C3aR axis regulates inflammatory gene expression, energy metabolism, and inflammation-associated memory [48]. In pancreatic β-cells, intracellular C3 interacts with the autophagy regulator ATG16L1 to enhance stress resilience [49]. Notably, these intracellular complement processes operate independently of the classical extracellular complement cascade and are now recognized as critical regulators of immune homeostasis, stress adaptation, and inflammation resolution. Dysregulation of intracellular C3 activity has been increasingly implicated in chronic inflammatory conditions, including vascular diseases, highlighting the complosome as a novel and essential layer of complement biology [22].

## Complement C3 functions in inflammation and vascular remodeling

Vascular remodeling refers to structural and functional alterations of the vessel wall that occur in response to various stimuli, such as hemodynamic overload, inflammation, or metabolic disturbances [50,51]. These changes involve modifications in lumen diameter, wall thickness, extracellular matrix (ECM) composition, and cellular constituents. While vascular remodeling can be adaptive under certain conditions, it often manifests as pathological in diseases such as hypertension, atherosclerosis, and aneurysms [52,53]. The process is initiated at the cellular level through imbalances in function and intercellular interactions. Within the intima, endothelial dysfunction triggers inflammatory responses and compromises barrier integrity [54]. VSMCs, under pathological cues, undergo phenotypic switching from a contractile to a synthetic state, characterized by increased migration, proliferation, and ECM production [55,56]. In the adventitia, fibroblasts and stromal cells are activated by inflammatory and immune-related signals, promoting ECM deposition and fibrosis [57]. Concurrently, innate immune cells (e.g., neutrophils, monocytes/macrophages) and adaptive immune cells (T and B lymphocytes) are recruited and activated, amplifying local inflammation [55]. Platelets and the coagulation cascade further contribute by reinforcing the interplay between inflammation and thrombosis [58,59]. The sustained cross-talk among these diverse cell types collectively drives the progressive alterations in vascular structure and function [55].

**Figure 2:**
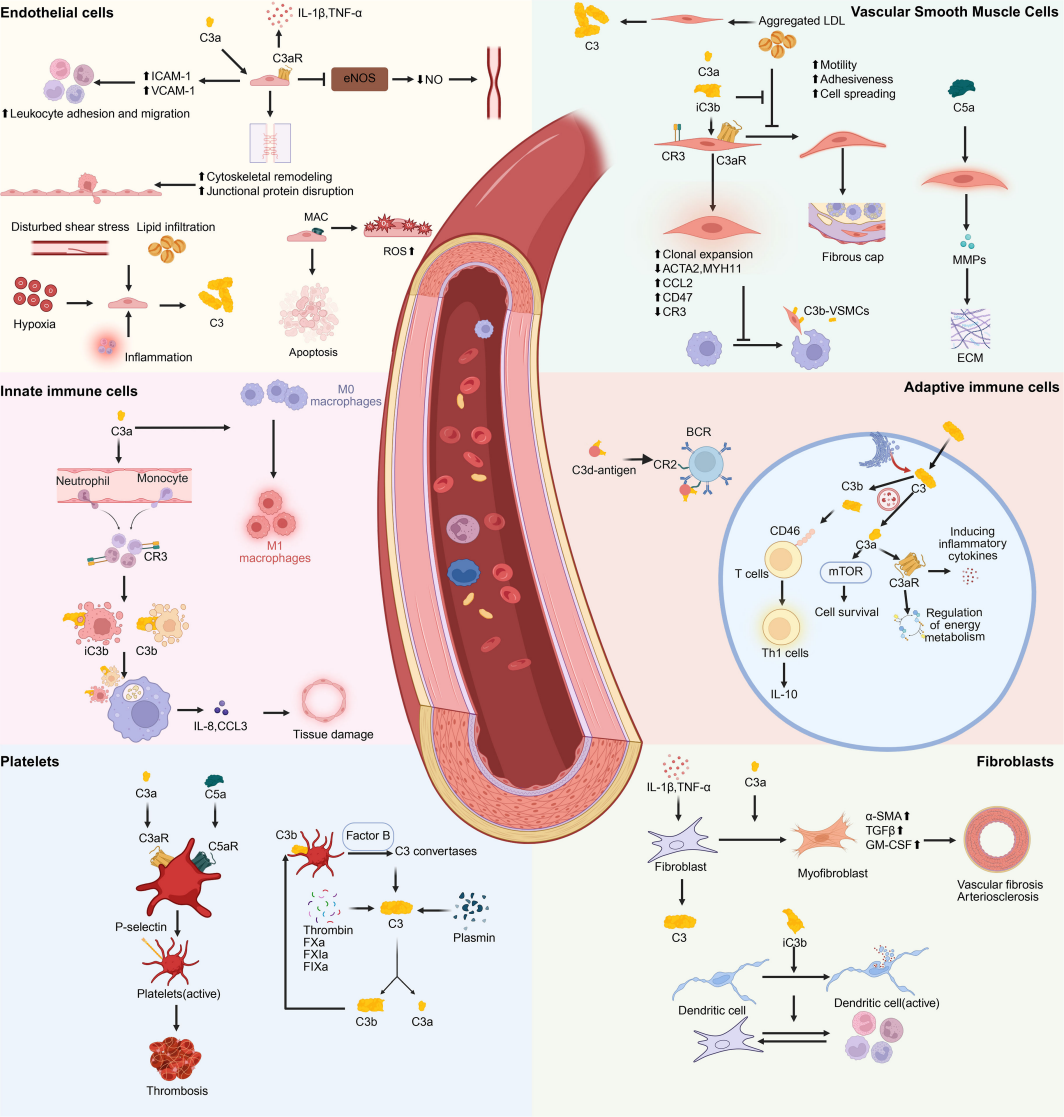
Cell-type-specific roles of C3 in vascular inflammation and remodeling. Complement C3 regulates immune–vascular interactions across multiple cell types. In endothelial cells, C3a/C3aR signaling promotes pro-inflammatory cytokine production, leukocyte adhesion, cytoskeletal disruption and reduced nitric oxide synthesis. In VSMCs, C3a and iC3b drive phenotypic switching, proliferation and fibrous cap formation, while C5a induces matrix degradation via MMPs. In innate immune cells, C3 fragments enhance neutrophil and monocyte recruitment, promote CR3-mediated phagocytosis and M1 macrophage polarization. In adaptive immune cells, intracellular C3 signaling regulates T cell activation and metabolic reprogramming, while C3d enhances B cell co-stimulation. Platelet-bound C3a and C5a promote activation and thrombosis. In fibroblasts, C3a induces myofibroblast transition and fibrosis, whereas iC3b supports dendritic cell activation. ACTA2, actin alpha 2; BCR, B-cell receptor; C3aR, C3a receptor; C5aR, C5a receptor; CCL2, C-C motif chemokine ligand 2; CD46, cluster of differentiation 46; CD47, cluster of differentiation 47; CR2, complement receptor 2; CR3, complement receptor 3; ECM, extracellular matrix; eNOS, endothelial nitric oxide synthase; FIXa, factor IXa; FXa, factor Xa; FXIa, factor XIa; GM-CSF, granulocyte-macrophage colony-stimulating factor; CAM-1, intercellular adhesion molecule-1; IL-10, interleukin-10; LDL, low-density lipoprotein; MAC, membrane attack complex; MMPs, matrix metalloproteinases; mTOR, mechanistic target of rapamycin; MYH11, myosin heavy chain 11; NO, nitric oxide; P-selectin, platelet selectin; ROS, reactive oxygen species; TGF-β, transforming growth factor-beta; Th1, T helper type 1 cell; VCAM-1, vascular cell adhesion molecule-1; α-SMA, alpha-smooth muscle actin.

As the central hub of all three complement activation pathways, C3 plays a pivotal role in bridging innate immune sensing with vascular pathology [17]. Upon activation, C3 generates effector fragments that amplify inflammation and initiate downstream cascades, transforming early immune responses into structural tissue injury [60]. Beyond its classical role in immune defense, C3 exerts diverse effects on various cell types involved in vascular remodeling, forming a cell-specific regulatory network that governs vascular permeability, immune cell recruitment, and tissue remodeling [22,26] (Figure 2).

### C3 and endothelial cells

Endothelial cells serve as both sentinels and effectors within the vascular system, forming a critical barrier between circulating blood and underlying tissues. They are not only highly responsive to complement activation but can also locally synthesize complement components, including C3, particularly under conditions of inflammation, hypoxia, or oxidative stress [61,62]. Upon activation, C3a binds to C3aR expressed on endothelial cells, initiating a cascade of pro-inflammatory signaling events [63]. These include increased expression of adhesion molecules such as ICAM-1 and VCAM-1, up-regulation of chemokines, and the promotion of leukocyte adhesion and transmigration [64–66]. Additionally, C3a also disrupts endothelial tight junctions, increasing vascular permeability and contributing to barrier dysfunction in sensitive vascular beds such as the brain, kidney, and lung [67–69].

C3b deposition on endothelial surfaces further amplifies local complement activity through the formation of alternative pathway convertases. This can lead to sublytic complement attack, characterized by oxidative stress, reactive oxygen species (ROS) generation, and endothelial apoptosis [70–72]. These effects, when transient and tightly regulated, support immune surveillance and facilitate tissue repair by permitting controlled immune cell access. However, under sustained or dysregulated conditions, the endothelial response may shift from protective to pathogenic. Persistent complement activation can disrupt vascular integrity and promote maladaptive remodeling, contributing to chronic microvascular injury, fibrosis, and tissue ischemia [73].

### C3 and vascular smooth muscle cells

VSMCs are essential for maintaining vascular tone and structural integrity under physiological conditions [74]. However, under pathological stimuli, such as oxidative stress, pro-inflammatory cytokines, or lipid accumulation, VSMCs undergo phenotypic transition and actively contribute to inflammation and vascular remodeling [75]. Recent studies have demonstrated that VSMCs not only express complement receptors, including C3aR and CR3, but can also produce C3 locally, particularly in response to cytokines or within atherosclerotic lesions [75–77]. This suggests that complement signaling functions in an autocrine or paracrine manner to influence VSMC behavior within inflamed or damaged vascular regions.

C3a-C3aR signaling has been shown to induce the phenotypic transition of VSMCs from a contractile to a synthetic, pro-inflammatory state, characterized by enhanced proliferation, migration, and ECM production, hallmarks of vascular remodeling in chronic inflammatory diseases [78]. In parallel, C5a signaling can up-regulate matrix metalloproteinases (MMPs), promoting ECM degradation and contributing to plaque instability in advanced atherosclerosis [79,80]. Furthermore, C3a deposition within the vascular intima promotes macrophage recruitment and activation, which in turn release cytokines and growth factors that further stimulate VSMC activation and vessel wall thickening [64]. Collectively, these complement-mediated processes establish a localized feedback loop of inflammation and tissue remodeling, accelerating intimal hyperplasia, vascular stiffening, and the progression of atherosclerotic and fibrotic disease. This pathogenic axis is increasingly recognized not only in coronary artery disease but also in peripheral and cerebrovascular beds, highlighting its relevance to panvascular pathology.

### C3 and innate immune cells

C3 plays a central role in recruiting and modulating the function of innate immune cells, particularly neutrophils and monocytes, during vascular inflammation [81]. The C3 cleavage product C3a, along with C5a, acts as a potent chemoattractant, guiding these cells to migrate to sites of vascular damage [82]. Upon tissue infiltration, neutrophils and monocytes interact with opsonins such as C3b and iC3b, which are deposited on apoptotic cells, pathogens, or damaged endothelium [83]. Recognition of iC3b through CR3 (CD11b/CD18) facilitates phagocytosis and triggers the release of pro-inflammatory cytokines and chemokines, including IL-8 and CCL3, thereby amplifying local inflammation and endothelial activation [15,84].

In macrophages, C3 fragments contribute to phenotypic polarization. Engagement of C3b with CR3 and autocrine C3a-C3aR signaling promotes skewing toward a pro-inflammatory, M1-like phenotype characterized by increased production of TNF-α, IL-1β, and inducible nitric oxide synthase (iNOS) [29,85,86]. This polarization enhances the local inflammatory milieu within the vessel wall, sustaining immune activation and promoting vascular remodeling. Additionally, complement-opsonized immune complexes can further stimulate macrophage inflammasome activation, contributing to tissue damage in chronic inflammatory states [87].

### C3 and adaptive immune cells

Upon activation, T cells autonomously express and process C3, forming a ‘self-complement’ circuit. Intracellular C3 is cleaved by lysosomal enzymes like cathepsin L into C3a and C3b, which bind to intracellular C3aR and CD46 to modulate T cell fate and function [46]. The C3a-C3aR axis activates the mTOR signaling pathway, enhancing glucose uptake and metabolic reprogramming to meet the demands of T cell activation. The C3b-CD46 axis regulates the differentiation of CD4^+^ T cells into Th1 cells and induces IL-10 production in the late stages of activation, establishing a time-dependent switch from inflammation to tolerance [88]. Other receptors, such as CR1 and CR3, stabilize the immunological synapse between antigen-presenting cells and T cells, improving antigen-presentation efficiency. C3 signaling significantly influences T cell polarization: signals from C3aR and C5a receptor (C5aR) favor Th1/Th17 differentiation, while the absence of these signals skews T cell fate towards regulatory T cells (Tregs) [89,90]. In infectious or tumor microenvironments, C3 signaling can shape or reverse the exhausted T cell (Tex) phenotype [91]. In chronic vascular inflammation, such as in atherosclerosis or vasculitis, dysregulated C3 signaling in T cells may favor persistent Th1 or Th17 polarization and contribute to vascular immune pathology [29,92,93].

The C3 cleavage fragment C3d serves as a crucial link between innate recognition and adaptive humoral immunity. By binding to complement receptor 2 (CR2/CD21) on B cells, C3d provides a co-stimulatory signal that synergizes with B cell receptor (BCR) signaling, significantly lowering the activation threshold [94–96]. During the primary immune response, C3b deposited on antigen surfaces is further processed into C3dg and C3d, which remain stably attached. These antigen-C3d complexes are captured by follicular dendritic cells and presented to B cells. B cells engage both BCR and CR2 to form immune synapses [97]. This dual engagement enhances B cell activation, promotes IL-4/IL-6-dependent proliferation, facilitates somatic hypermutation and affinity maturation, and supports the formation of memory B cells and long-lived plasma cells [16,94]. In vascular contexts, this may contribute to autoantibody production or complement-mediated immune complex deposition.

### Platelets and coagulation cells

C3 also interacts closely with the hemostatic system, particularly platelets and coagulation factors, establishing a direct link between vascular inflammation and thrombosis [98]. Platelets express receptors for C3a and C5a, and exposure to these anaphylatoxins enhances platelet activation, aggregation, and surface expression of pro-coagulant molecules such as P-selectin and phosphatidylserine [99,100]. These changes not only promote thrombus formation but also facilitate further complement activation at the platelet surface.

C3b can deposit on activated platelets and microparticles, contributing to the formation of alternative pathway convertases and propagating the complement cascade [99]. In turn, activated platelets can serve as platforms for local C3 cleavage, creating a feedback loop between complement and coagulation [101]. Complement-induced platelet activation has been implicated in both arterial thrombosis (e.g., myocardial infarction, stroke) and microvascular thrombosis in diseases such as antiphospholipid syndrome, thrombotic microangiopathy, and COVID-19–associated coagulopathy [102–104].

Moreover, several coagulation and fibrinolytic enzymes, including thrombin, FXa, FXIa, FIXa, and plasmin, can directly cleave C3 into C3a and C3b, acting as non-canonical C3 convertases that initiate or amplify complement activation under inflammatory or prothrombotic conditions [29,38]. This bidirectional cross-talk integrates immune surveillance with hemostatic regulation but, when dysregulated, contributes to a pro-inflammatory and pro-thrombotic vascular environment.

### C3 and fibroblasts and stromal cells

Fibroblasts and vascular stromal cells, traditionally regarded as structural elements, are now recognized as active modulators of immune responses and chronic vascular inflammation [105]. In response to inflammatory cytokines (e.g., IL-1β, TNF-α), pathogen-associated molecular patterns (e.g., lipopolysaccharide), or mechanical stress, fibroblasts in the adventitia and perivascular regions up-regulate the expression of complement components, including C3 (106–108). This localized complement production occurs independently of liver-derived sources and contributes to the establishment of a tissue-specific pro-inflammatory milieu.

C3a signaling in fibroblasts promotes a shift toward a myofibroblast phenotype characterized by increased α-SMA expression, ECM deposition, and secretion of fibrogenic cytokines such as transforming growth factor-beta (TGF-β) and chemokine such as GM-CSF [109]. This phenotype is central to the development of perivascular fibrosis and arterial stiffening, which are common features of chronic vascular diseases including hypertension, heart failure, and renal fibrosis [109,110]. Furthermore, iC3b and C3d fragments can modulate fibroblast–immune cell interactions by influencing dendritic cell maturation and macrophage activation in the stromal compartment [27,111]. In barrier-rich tissues such as the lung, gut, and kidney, stromal cells contribute to a local C3-driven immunological niche, shaping both inflammation and tissue repair. Persistent C3 activation in these settings may impair resolution and drive fibrotic remodeling, reinforcing the link between complement dysregulation and vascular tissue pathology [69,112,113].

Together, the cell-type-specific functions of C3 position it as a central integrator of immune–vascular cross-talk. When dysregulated or persistently activated, C3 drives a pathological cascade involving endothelial injury, immune cell infiltration, thromboinflammation, and fibrotic remodeling. This convergence of mechanisms across diverse cellular compartments underscores C3’s pivotal role in the pathogenesis of panvascular diseases, where distinct vascular beds are affected by shared inflammatory and structural insults. These insights not only reinforce the biological importance of C3 but also highlight its promise as a therapeutic target for modulating vascular inflammation in a system-wide, disease-agnostic manner.

## C3-mediated mechanisms in panvascular diseases

**Figure 3:**
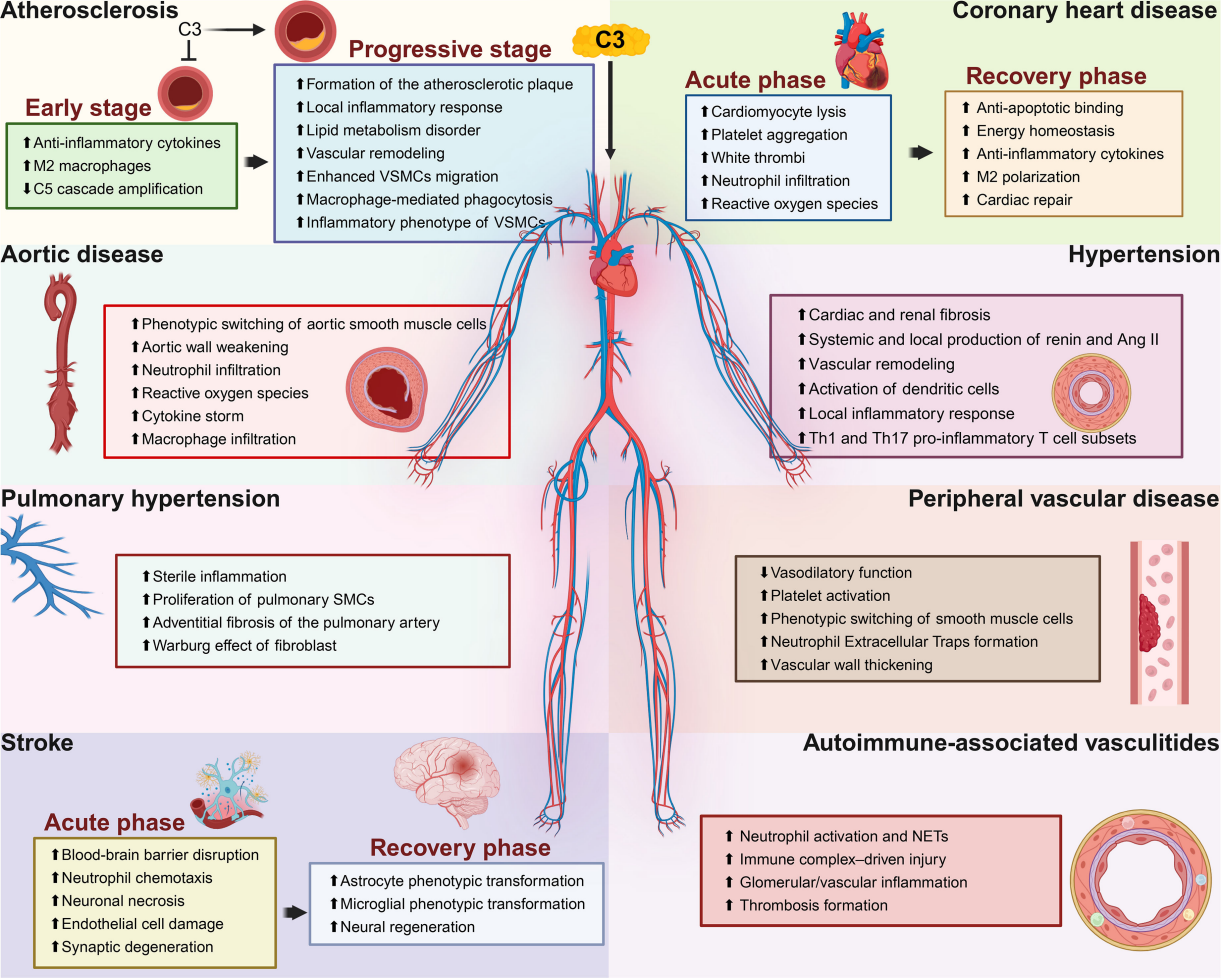
The role of C3 in panvascular disease. Complement C3 contributes to the pathogenesis of a broad spectrum of panvascular diseases by modulating immune–vascular interactions in a disease- and context-specific manner. In atherosclerosis, C3 exerts dual effects depending on disease stage: early activation promotes anti-inflammatory cytokine production, M2 macrophage polarization and limits downstream C5 activation, whereas sustained activation in the progressive stage drives plaque development, vascular remodeling, lipid metabolic dysfunction, VSMC migration and inflammation. In coronary heart disease, C3 shows phase-dependent effects: in the acute phase it aggravates injury by promoting cardiomyocyte lysis, platelet aggregation, white-thrombus formation, neutrophil infiltration, and reactive oxygen species generation; in the recovery phase it supports repair by enhancing anti-apoptotic signaling, maintaining energy homeostasis, increasing anti-inflammatory cytokine production, and driving M2 macrophage polarization and cardiac repair. In aortic disease, C3 drives SMC dedifferentiation, wall weakening, neutrophil and macrophage infiltration, oxidative stress, and cytokine storm. In hypertension, C3 is associated with increased cardiac and renal fibrosis, enhanced RAS activation, vascular remodeling, dendritic cell activation, and accumulation of Th1 and Th17 inflammatory T cell subsets. In pulmonary hypertension, C3 promotes sterile vascular inflammation, proliferation of pulmonary SMCs, fibroblast metabolic reprogramming (Warburg effect), and adventitial fibrosis. In peripheral vascular disease, C3 contributes to impaired vasodilatory function, platelet activation, phenotypic switching of VSMCs, NETs formation and vascular wall thickening. In stroke, C3 exacerbates acute brain injury by promoting blood-brain barrier disruption, neutrophil chemotaxis, neuronal necrosis, endothelial cell damage and synaptic degeneration, while in the recovery phase it supports neural regeneration through phenotypic transformation of astrocytes and microglia. In autoimmune-associated vasculitides, C3 links autoimmunity to vascular injury by promoting neutrophil activation and NETs formation, driving immune-complex–mediated damage, amplifying glomerular/vascular inflammation, and facilitating thrombosis formation. Ang II, angiotensin II; SMCs, smooth muscle cells; NETs, neutrophil extracellular traps; Th1/Th17, T helper 1/17 cells; VSMCs, vascular smooth muscle cells.

Building upon its fundamental roles in inflammation and vascular permeability, complement C3 contributes to the pathogenesis of panvascular diseases by mediating immune activation and tissue remodeling across multiple vascular beds (Figure 3, Table 2).

**Table 2:** C3-mediated mechanisms in panvascular diseases

Diseases	Target cells/tissues	Mechanisms	Pathological consequences	References
Atherosclerosis	Macrophages	C3b binds to oxidized LDL promoting foam cell formation	Accelerates plaque progression	[64,114]
		iC3b forms regulatory complexes with CFH to enhance necrotic cell clearance	Early protective effects against hyperlipidemic injury	[75,115]
	Endothelial cells	C3a up-regulates ICAM-1/VCAM-1 via C3aR	Disrupts vascular barrier integrity	[64,116]
		Promotes IL-1β/TNF-α release	Enhances monocyte infiltration	[65]
	Smooth muscle cells	Induces synthetic phenotype transition	Dual regulation: stabilizes plaque structure while promoting clonal expansion	[75]
		Stimulates collagen secretion and fibrous cap formation		[117]
Coronary artery disease	Cardiomyocytes	Ischemic phase: C3-cytochrome C complexes inhibit apoptosis	Acute phase: expands infarct area	[118,119]
		Reperfusion phase: complement cascade activates NLRP3 inflammasome	Chronic phase: affects myocardial repair and scar maturation	[120]
	Platelets	C3a synergizes with GPIIb/IIIa to promote Ca²⁺ influx	Facilitates coronary white thrombus formation	[121]
Aortic diseases (Aneurysm/Dissection)	Vascular media	C3a-C3aR axis activates MMP2 secretion	Elastic fiber degradation	[122]
		Alternative pathway dominates local complement activation	70% reduction in dissection incidence (mouse model)	[123]
	Immune cells	C3a induces NF-κB signaling in macrophages	Promotes inflammatory microenvironment in vascular wall	[122]
		3–5 fold increase in CD11b⁺ cell infiltration		[24]
Hypertension	Renal tissue	C3a stimulates renin secretion	20–30mmHg blood pressure elevation (SHR model)	[125]
		Regulates angiotensinogen expression via KLF5/LXRα	Renal interstitial fibrosis	[126]
	Perivascular adipose tissue	Bone marrow-derived macrophage C3 deposition	Impairs vascular relaxation	[127]
		Suppresses adiponectin secretion	Enhances endothelium-dependent contraction	[128]
Pulmonary arterial hypertension	Pulmonary artery fibroblasts	Intracellular complosome regulates glycolysis	45% increase in pulmonary vascular resistance	[108]
		Secretes IL-6/IL-33 promoting adventitial fibrosis	Right ventricular hypertrophy	[29]
	Pulmonary vascular macrophages	C3a induces NLRP3 inflammasome assembly	50-80% vascular wall thickening	[129]
		IL-1β promotes PASMC proliferation	Elevated mean pulmonary arterial pressure	
Peripheral vascular disease	Vascular endothelium	C3a inhibits eNOS activity (40% NO reduction)	30% reduction in lower limb blood flow	[130]
		Enhances P-selectin expression	Intermittent claudication	[131]
	Platelets	PI3K-Akt-Rap1b signaling pathway activation	Increased risk of acute limb ischemia	[132]
		2-fold increase in platelet aggregation rate		
Stroke	Blood-brain barrier	C3a-C3aR disrupts tight junction proteins	35-50% expansion of infarct volume	[68,133]
		Enhances neutrophil transendothelial migration	Hemorrhagic transformation	[63]
	Neuroglial cells	Acute phase: Microglial CR3 mediates synaptic phagocytosis	Cognitive impairment	[134,135]
		Recovery phase: C3a promotes Thbs4 secretion by astrocytes	Promotes neural network reorganization after 7 days	[133]
Autoimmune-associated vasculitides	Glomeruli	Prominent local synthesis and deposition	Worse renal function outcomes	[136]
	Platelets	Decreased C3 is associated with thrombocytopenia.	Abnormal coagulation function	[137,138]

### Atherosclerosis

Atherosclerosis, the leading cause of cardiovascular and cerebrovascular diseases, is increasingly recognized as a chronic inflammatory condition driven by lipid dysregulation and innate immune activation [139]. Among innate immune pathways, the complement system has emerged as a key mediator of vascular inflammation, foam cell formation, VSMC phenotypic switching, and endothelial dysfunction [114,140,141]. Multiple population-based and clinical studies support the role of C3 as a cardiovascular risk factor [116,142]. Elevated plasma C3 levels are associated with hypertension, obesity, dyslipidemia, and increased risk of myocardial infarction[72,117]. In the CODAM study, higher C3 and C3a levels correlated with carotid intima-media thickness, dyslipidemia, and cardiovascular risk, particularly among smokers[61,72,116]. C3a levels were also independently associated with subclinical atherosclerosis and peripheral arterial disease [116]. High intraplaque expression of C3/iC3b, identified through proteomic analyses, predicted cardiovascular events after endarterectomy and outperformed macrophage density and neovascularization scores [75]. Properdin, a positive regulator of the alternative pathway, showed similar associations with plaque vulnerability and cardiovascular mortality [143].

Mechanistically, C3 activation occurs early in atherogenesis and primarily facilitates immune recognition and non-inflammatory clearance during this stage. Pattern recognition molecules such as C-Reactive protein (CRP) and natural IgM antibodies bind oxidized LDL (oxLDL) in the arterial wall, initiating classical and alternative pathway activation, while detection of MBL and ficolins in plaques supports lectin pathway involvement [114,144]. Cholesterol crystals, another early trigger, robustly activate all three complement pathways and generate abundant C3 fragments [145]. Among them, C3a and iC3b may rescue VSMCs from aggregated LDL-induced dysfunction, promoting cytoskeletal remodeling and fibrous cap formation, a protective adaptation during early plaque development [75]. Meanwhile, C3a, through C3aR signaling, activates endothelial cells and macrophages to produce IL-1β and TNF-α, facilitating a controlled inflammatory response [116,146]. In macrophages, C3b opsonizes oxLDL, facilitating lipid uptake, while C3b/iC3b engagement with CR3/CR4 promotes efferocytosis of apoptotic cells and oxidized lipids, helping preserve immune homeostasis [114,147]. CR3 signaling downstream of Syk and PI3K supports actin remodeling and phagocytosis and can induce anti-inflammatory IL-10 production and M2 macrophage polarization upon co-stimulation with fibronectin [115]. Supporting this, C3-deficient mice exhibit greater lipid retention, apoptotic burden, and unstable plaque features with increased macrophages and reduced VSMCs and collagen [115]. These findings suggest that regulated C3 activation exerts protective effects in early atherosclerosis.

As atherosclerosis advances, the balance shifts. Macrophages continuously ingest oxLDL and undergo apoptosis; however, under conditions of complement imbalance, opsonin depletion, and elevated ‘don’t eat me’ signals such as CD47 impair apoptotic cell clearance, leading to their accumulation, necrotic core expansion, and plaque destabilization [115,117]. Infiltrating immune cells and vascular cells locally synthesize C3 and its regulators, further amplifying complement activation via autocrine and paracrine routes [114,147]. Co-expression of C3 and CFH in macrophages and high C3/iC3b deposition in VSMC-rich plaque areas confirm local complement activity [75,115]. Lipid-loaded VSMCs also express and secrete C3/C3a, contributing to lesion remodeling [75]. VSMCs are considered key structural cells in forming and stabilizing the fibrous cap, undergoing phenotypic switching, clonal expansion, and adopting inflammatory, immune-like features during atherogenesis [117]. These modifications cause VSMCs to up-regulate the anti-phagocytic marker CD47 and down-regulate CR3, rendering them resistant to phagocytosis even in the presence of substantial C3b deposition [115,117]. Similarly, endothelial cells up-regulate C3 and C3aR in response to disturbed shear stress and lipid exposure, becoming active sources of local complement and amplifying inflammatory signaling [114,148]. Intracellular complement activation, via self-synthesis and cleavage of C3, followed by C3aR engagement on lysosomal or mitochondrial membranes, also modulates metabolism and function in both endothelial and immune cells [115]. Sustained C3 activation exacerbates inflammation and lesion progression. C3-deficient mice show reduced plaque burden and inflammation in high-fat diet models, and deletion of factor B attenuates plaque size and complexity, highlighting the pathogenic contribution of uncontrolled complement activity [149]. Pharmacological inhibition of C3aR reduces M1 macrophages and favors M2 polarization, while genetic C3 deletion similarly promotes an anti-inflammatory shift [147].

In summary, C3 in atherosclerosis displays strong cell-type specificity and dual functionality. In endothelial cells, C3a-C3aR signaling drives chemotaxis and inflammation but also supports metabolic and barrier integrity under homeostasis [114,147]. In macrophages, C3b/iC3b promotes efferocytosis and tolerance, whereas dysregulation skews toward inflammation and necrotic core expansion [115,147]. In VSMCs, C3/C3a signaling drives remodeling and immune evasion, contributing to both cap stability and plaque complexity [75,117]. Thus, C3 functions as a context-dependent immunometabolic regulator rather than a unidirectional effector. Recognizing this duality paves the way for therapeutic strategies targeting specific complement functions or cellular contexts rather than broad inhibition.

### Coronary heart disease

Coronary heart disease (CHD), including myocardial infarction (MI) and ischemia-reperfusion (I/R) injury, remains a leading contributor to cardiovascular mortality [150]. Beyond lipid dysregulation and thrombosis, inflammation plays a pivotal role in myocardial damage, with the complement C3 emerging as a central player in both injury and recovery [151]. Compelling clinical data support activation of the complement system in patients with MI [152,153]. Epidemiological studies have demonstrated that elevated serum C3 correlates with traditional CHD risk factors such as obesity, hypertension, dyslipidemia, and hyperglycemia [154,155]. Notably, individuals in the highest tertile for serum C3 had a tenfold increased risk of future MI, independent of other inflammatory markers like CRP [154,155].

Transcriptomic analyses further confirm complement activation at the molecular level in acute MI. Compared with healthy individuals and patients with stable angina, those with acute MI show up-regulation of classical pathway components (C1qα/β/γ), properdin, C5a, and their receptors (e.g., C5aR, CR1), along with decreased expression of MAC components (C7, C8β, C9) [151,152]. Increased serum levels of total C3, C4, and CH50 have also been consistently observed [152]. In the setting of MI, coronary artery occlusion leads to profound ischemia. While reperfusion therapy is essential for myocardial salvage, reintroduction of oxygenated blood paradoxically exacerbates tissue damage through a robust inflammatory cascade [156]. One of the earliest responses to ischemic necrosis is the release of damage-associated molecular patterns (DAMPs), which activate the classical and lectin pathways of complement, rapidly generating C3 cleavage products [118,157]. Experimental models show that C3 deposition concentrates at the infarct border zone, as detected by radiolabeled C3d probes, coinciding with regions of peak inflammation [158]. The resultant C3a and C5a fragments initiate a cascade of events: neutrophil chemotaxis, degranulation, ROS release, and mast cell activation, all of which contribute to further cardiomyocyte injury [118,152]. MAC formation adds another layer of cytotoxicity by inducing cardiomyocyte lysis. Pharmacologic and genetic interventions confirm these effects [159]. Inhibiting complement activation via soluble CR1 (sCR1) or the endogenous inhibitor MAP-1 reduces infarct size and neutrophil infiltration, underscoring the pathogenic role of uncontrolled C3 activation in acute phases [130].

Beyond inflammation, complement also interfaces with thrombosis, a key feature of MI pathogenesis. C3a, through binding to C3aR on platelets, enhances calcium influx, adhesion, and aggregation, especially when acting synergistically with glycoprotein IIb/IIIa [121]. These actions facilitate the formation of white thrombi and increase the risk of reocclusion following reperfusion.

In contrast to its pro-inflammatory reputation, recent findings have uncovered a protective, intracellular role for C3 in cardiomyocytes. In murine I/R models, C3-deficient (C3⁻/⁻) mice displayed smaller infarcts than wildtype controls, partly due to reduced necrotic expansion [120]. Mechanistically, C3 protein was shown to bind to cytochrome c and pro-caspase-3 within cardiomyocytes, preventing activation of the apoptotic cascade [120]. *In vitro,* exogenous C3 addition attenuated cardiomyocyte apoptosis under hypoxic stress [120]. Moreover, C3 modulates cardiomyocyte metabolism [160]. In isolated perfused heart models, C3⁻/⁻ hearts exhibited disrupted NAD^+^/NADH homeostasis during early reperfusion, implicating intracellular C3 in regulating energy recovery and redox balance [160].

Beyond acute injury, C3 also shapes long-term myocardial healing. In non-reperfused MI models, C3-deficient mice experienced worsened ventricular dilation and impaired cardiac function during chronic recovery [119]. This suggests that C3 signaling contributes to myocardial preservation, possibly by facilitating scar formation, immune resolution, or progenitor cell activation. C3 may also modulate immune cell polarization. Pharmacological blockade of C3aR favors an anti-inflammatory M2 macrophage phenotype, while genetic C3 deletion similarly skews immune balance toward tissue repair [161,162]. These effects highlight that C3, depending on timing and cellular context, may either exacerbate damage or facilitate resolution.

### Aortic diseases

Aortic diseases encompass a spectrum of pathologies involving the structural and functional compromise of the aorta, including thoracic aortic aneurysm (TAA), abdominal aortic aneurysm (AAA), and aortic dissection (AD). Their pathogenesis is multifactorial, involving VSMCs apoptosis, ECM degradation, oxidative stress, immune infiltration, and chronic inflammation [163,164]. Emerging evidence identifies complement C3 as a critical mediator in the initiation and progression of these lesions [122]. In patients with thoracic aortic dissection (TAD) and TAA, circulating levels of C3a, C4a, and C5a are significantly elevated during the acute phase and correlate with increased risk of rupture [123]. Similarly, in AAA, systemic C3 levels decrease in advanced cases while tissue C3 deposition intensifies in the thrombus and vascular wall, revealing a focal ‘systemic down-regulation–local enrichment’ imbalance [165]. In TAA, transcriptomic analyses have revealed up-regulation of C3 and C3aR in diseased tissues, with these genes emerging as central hubs in co-expression networks related to vascular remodeling and inflammation [166].

Mechanistic insights from animal models further support a pathogenic role of C3 in aortic disease. In TAD, β-aminopropionitrile-induced models exhibit rapid elevation of C3a and up-regulated C3aR expression in medial SMCs at sites of damage [122]. Genetic deletion of C3aR reduces dissection rates and CD45^+^ cell infiltration, highlighting its role in promoting both inflammation and wall destabilization [122]. Functionally, C3a stimulates SMCs to secrete MMP2 via C3aR signaling, directly contributing to ECM degradation. Moreover, knockout of the Cfb gene significantly reduces C3a/C5a generation and immune cell recruitment, emphasizing the predominance of the alternative pathway in TAD pathogenesis [123].

In AAA, up-regulation of C3 has been confirmed in aneurysmal tissues, with transcription factor STAT5A implicated in co-ordinated regulation [167]. Elastase-induced mouse models show that C3a and C5a promote neutrophil infiltration, ROS production, and MMP9 expression, driving medial degeneration [168]. These findings align with observations that C3 induces ROS generation by polymorphonuclear neutrophils (PMNs) [165]. Complement depletion using cobra venom factor significantly reduces AAA formation and aortic diameter, and C3a and C5a antagonists block chemotactic responses [169]. C3 also regulates adaptive responses by modulating the phenotype and recruitment of macrophages, CD4^+^ T cells, and B cells in the aneurysmal microenvironment [169].

In TAA, co-expression of C3, C3aR, and chemokine receptor CX3CR1 suggests that C3 signaling facilitates immune cell recruitment through induction of chemokines such as CX3CL1(150). Complement activation fragments not only guide immune cell migration but also enhance the release of TNF-α, IL-1β, IL-6, and MCP-1. In human surgical models, early postoperative surges in complement activation products (C4bc, C3bc, TCC) correlate with pro-inflammatory cytokine levels and are temporally synchronized with fluctuations of post-inflammatory regulators such as IL-6 and IL-10 [170]. In macrophages, C3a and C5a activate the TIAM1-RAC1 axis, triggering (nuclear factor-kappa B) NF-κB signaling and large-scale cytokine production including MMPs and IL-6 [155]. In parallel, SMCs respond to C3a through autocrine and paracrine C3aR engagement, activating NF-κB and AP-1 pathways that elevate MMP2 expression and promote ECM degradation. This structural remodeling, independent of immune cell-mediated inflammation, exacerbates wall fragility and predisposes to dissection.

In summary, complement C3 acts as a central co-ordinator of immune–vascular interactions in aortic diseases. By orchestrating immune cell recruitment, promoting smooth muscle cell reprogramming, and enhancing matrix degradation, C3 actively contributes to the inflammatory and structural deterioration of the aortic wall. These findings position C3 as a key mechanistic bridge linking immune activation to progressive vessel wall remodeling and destabilization in pan-aortic pathology [171].

### Hypertension

Hypertension is a major global health burden characterized by sustained elevation of arterial pressure and progressive damage to target organs. Its multifactorial pathogenesis involves complex interactions among the vascular, renal, nervous, and endocrine systems [172]. Recent studies highlight chronic inflammation and immune dysregulation as key drivers, with complement C3 emerging as a central mediator linking immune activation, vascular remodeling, and hormonal imbalance [173,174]. Aberrant C3 expression has been observed in hypertensive vasculature, kidneys, and adipose tissue [125–127,175]. Its activation products, C3a and C3b, promote immune cell polarization, vascular remodeling, renal dysfunction, and local renin-angiotensin system (RAS) activation, collectively driving hypertension onset and progression [173,176].

Clinical studies have consistently linked complement C3 dysregulation to the development and progression of hypertension. Elevated plasma C3 levels have been observed in hypertensive patients and are accompanied by increased immunoglobulin titers and reduced circulating T cell subsets, indicating systemic immune activation and imbalance [177–179]. In a 2024 case-control study, Wang *et al*. demonstrated a stepwise increase in plasma C3 and the alternative pathway activation marker AP50 across healthy controls, hypertensive individuals without renal impairment, and those with renal complications [180]. Conversely, levels of complement factor H (CFH), a negative regulator of the alternative pathway, declined progressively, reflecting a regulatory imbalance that may facilitate sustained C3 activation [180]. Importantly, multivariate analysis confirmed elevated C3 as an independent risk factor for both hypertension and hypertensive renal injury. These findings were reinforced by a large prospective cohort study, which identified baseline C3 levels as a significant predictor of future hypertension, independent of confounding metabolic parameters [179,181]. Beyond blood pressure elevation, C3 dysregulation is also associated with hypertension-related comorbidities such as metabolic syndrome, type 2 diabetes, and atherosclerosis, further highlighting its pathogenic relevance across cardiovascular and metabolic axis [182–184].

Findings from preclinical studies provide mechanistic insights into these associations. In spontaneously hypertensive rats (SHR), C3 expression is consistently elevated in plasma, VSMCs, renal tubulointerstitium, and glomeruli, reflecting systemic complement activation and local tissue engagement [125,175]. Functional depletion of C3 in SHR led to reduced blood pressure, suppressed renal renin expression and local RAS activity, decreased urinary catecholamines, and amelioration of renal interstitial fibrosis, suggesting that C3 drives both hormonal activation and target organ damage [125]. In a DOCA-salt-induced hypertension model, Ruan et al. reported prominent C3 deposition in perivascular adipose tissue (PVAT), primarily derived from bone marrow–derived macrophages, where it triggered local inflammation and vascular injury [127]. These findings collectively indicate that C3 acts across systemic and tissue-specific compartments to promote vascular dysfunction and immune activation in hypertension.

At the vascular level, C3 drives remodeling by modulating VSMC phenotype. Exogenous C3 exposure induces a transition from a contractile to a synthetic phenotype, characterized by DNA synthesis, SM22α down-regulation, and increased osteopontin and matrix Gla protein expression [126,175]. This transition is mediated by C3a-C3aR signaling, which activates the transcription factors KLF5 and LXRα, key regulators of VSMC proliferation and inflammation [126]. In SHR-VSMCs, treatment with C3 antisense oligonucleotides reverses this synthetic shift. Intriguingly, C3 overexpression is accompanied by suppression of miR-145, a microRNA that maintains contractile phenotype by inhibiting C3 expression. Loss of miR-145 leads to uncontrolled C3 induction, promoting synthetic transformation and up-regulation of proliferative cytokines such as TGF-β and PDGF-A [126]. In parallel, C3a-C3aR signaling in renal and vascular tissues also induces renin and angiotensin II synthesis [125,176]. Mechanistically, C3 enhances the expression of angiotensinogen and its transcriptional regulators KLF5 and LXRα in VSMCs. Supporting this, C3a has been shown to promote LXRα nuclear translocation and stimulate renin production during mesenchymal stem cell differentiation into smooth muscle-like cells, suggesting a role in establishing atypical renin-secreting phenotypes [176,185].

PVAT functions as a key immunometabolic niche in hypertension, and C3 plays a central role in its pro-inflammatory reprogramming. In C3-deficient mice, PVAT exhibits a favorable shift toward an anti-inflammatory profile, characterized by reduced M1 macrophage infiltration, increased M2 macrophages, and attenuated vascular injury [127,128]. This remodeling is partly driven by C5a, a downstream product of C3 cleavage, which promotes M1 polarization via C5aR and induces the expression of inflammatory mediators such as TNF-α, IL-6, iNOS, and MCP-1, while suppressing anti-inflammatory factors like adiponectin [127,128]. In parallel, both C3a and C5a enhance dendritic cell activation and skew T cell differentiation toward pro-inflammatory Th1 and Th17 lineages while suppressing regulatory T cells, thereby disrupting immune tolerance and exacerbating hypertensive end-organ damage [173,186].

In summary, complement C3 acts as a central mediator in hypertension by linking immune activation, vascular remodeling, and RAS dysregulation. Its co-ordinated effects across tissues and cell types drive both systemic inflammation and local vascular injury, positioning C3 as a key pathogenic factor in hypertensive disease progression.

### Pulmonary arterial hypertension

Pulmonary arterial hypertension (PAH) is a progressive vascular disorder marked by elevated pulmonary arterial pressure and vascular resistance, ultimately leading to right heart failure [187]. Central to its pathophysiology is the structural remodeling of distal pulmonary arteries, involving aberrant proliferation of smooth muscle cells (PASMCs), fibroblast activation, endothelial dysfunction, and ECM remodeling [188,189]. Recent evidence implicates complement C3 as a key regulator of these processes at both systemic and local levels [190,191]. In patients with idiopathic PAH (IPAH), elevated plasma C3 levels correlate with disease severity and cardiac dysfunction [191]. Complement activation products such as C3d are prominently deposited in perivascular regions of remodeled pulmonary arteries, especially within the adventitia and medial layers [129,190]. Single-cell transcriptomic analyses and immunohistochemistry have identified pulmonary fibroblasts as the dominant local source of C3 in IPAH [108]. These fibroblasts exhibit up-regulation of C3, CFB, and CFD at both transcript and protein levels, suggesting active local complement production independent of systemic sources [108].

Mechanistically, C3 contributes to PAH through both immune and structural pathways. In macrophages, C3a binds to C3aR to activate NF-κB signaling, ROS production, and calcium influx, leading to NLR family pyrin domain containing 3 (NLRP3) inflammasome assembly and caspase-1 dependent maturation of IL-1β and IL-18 [129]. These cytokines amplify sterile perivascular inflammation and promote PASMC proliferation by inducing PCNA and Cyclin D expression. Inhibition or genetic deletion of C3 blunts NLRP3 activation and attenuates downstream inflammatory and proliferative responses, supporting its pathogenic role in pulmonary vascular remodeling [129]. In parallel, pulmonary artery fibroblasts utilize intracellular complement signaling (the ‘complosome’) to regulate metabolism and matrix remodeling [110]. Upon activation, intracellular C3a binds to mitochondrial outer membrane-associated C3aR, shifting cellular metabolism toward aerobic glycolysis (Warburg effect) [192]. This metabolic reprogramming promotes the production of inflammatory cytokines (e.g., IL-6, IL-13, MCP-1, SDF-1, IL-33), enhances PASMC proliferation and macrophage recruitment, and activates TGF-β signaling. These changes collectively drive collagen and fibronectin deposition, contributing to adventitial fibrosis and long-term vascular stiffening [110,192].

Together, these findings position C3 as a central mediator in PAH pathogenesis. It links systemic inflammation to local vascular remodeling through both extracellular immune activation and intracellular metabolic reprogramming, with fibroblast-derived C3 at the nexus of this immuno-metabolic-fibrotic axis.

### Peripheral vascular disease

Peripheral vascular disease (PVD) encompasses structural and functional abnormalities of peripheral arteries, characterized by vascular wall remodeling, luminal narrowing, chronic ischemia, and a high risk of thrombosis [193]. Growing evidence implicates complement C3 as a critical mediator in these pathological processes. Large-scale population studies, such as the Maastricht Study involving 3019 individuals, have demonstrated that elevated plasma C3 levels are significantly associated with increased carotid-femoral pulse wave velocity and arterial wall stiffness [194]. This correlation persisted, though modestly, in type 2 diabetes patients even after multivariable adjustment [194]. These findings suggest a potential link between systemic C3 levels and peripheral arterial rigidity.

Mechanistically, C3 contributes to PVD through its effects on endothelial cells and VSMCs. C3a binding to endothelial C3aR up-regulates adhesion molecules like ICAM-1 and VCAM-1, enhances leukocyte adhesion and transmigration, and disrupts NO synthesis by inhibiting eNOS [130]. Additionally, C3a induces cytoskeletal changes and junctional disruption, increasing vascular permeability and facilitating access of inflammatory and thrombotic factors to the vessel wall [130]. In parallel, C3 promotes VSMC phenotypic switching toward a synthetic phenotype, characterized by down-regulation of SM22α and up-regulation of ECM proteins such as collagen types I and III and fibronectin, driving vascular stiffening and wall thickening [131].

As PVD progresses, arterial thrombosis becomes a life-threatening complication. Complement activation plays a crucial role in platelet priming and thrombus formation. Sauter *et al*. demonstrated that platelet surface expression of C3aR correlates with GPIIb/IIIa activation in patients with coronary artery disease, suggesting a role for C3a in platelet priming within high-risk populations [121]. In support, animal studies reveal that C3a binding to C3aR triggers platelet activation through the PI3K-Akt pathway to promote calcium influx and small GTPase Rap1b activation, enhancing platelet adhesion, spreading, and aggregation [121,132]. These effects are significantly attenuated in C3aR-deficient mice or upon C3aR blockade. Additionally, C3a promotes the formation of neutrophil extracellular traps (NETs), which provides a scaffold for thrombus development and activates intrinsic coagulation through recruitment of clotting factors. C3b, in parallel, binds to P-selectin and fibrin on activated platelets, enhancing complement deposition and reinforcing thrombus architecture [132,195].

Further supporting these findings, studies in venous thromboembolism (VTE) have observed elevated levels of complement factor H-related protein 5 (CFHR5), a regulator of the C3 alternative pathway, in patients with acute VTE. *In vitro,* recombinant CFHR5 enhances platelet activity and correlates with increased thrombin generation, pointing to a broader role of C3 pathway activation in thrombotic disorders [100].

Altogether, complement C3 functions as a central mediator of PVD by linking inflammatory activation, vascular remodeling, and thrombus formation. Its co-ordinated effects on endothelial integrity, VSMC phenotype, platelet function, and innate immunity underscore its relevance to peripheral vascular pathology and its potential as a therapeutic target.

### Stroke

Complement C3 plays a multifaceted and temporally dynamic role in stroke. Acting as a central immune mediator, C3 participates in both the acute inflammatory response and the long-term neurorepair processes. This biphasic behavior underpins its dual contribution to neuronal injury and recovery, making it a key node in post-stroke pathophysiology [196,197].

Clinically, elevated plasma C3 levels have been shown to predict worse 3 month neurological outcomes in patients with acute ischemic stroke [140,198,199]. Alawieh *et al*. demonstrated that circulating C3a and C3d levels positively correlate with stroke severity (NIHSS) and functional disability at discharge (mRS) [134,200]. Postmortem analyses identified extensive C3 and C3b deposition in synaptic zones of stroke patients, often co-localized with activated microglia and areas of synaptic loss. Moreover, C3aR expression is significantly up-regulated in astrocytes and microglia from stroke brains, and higher expression levels are associated with delayed motor recovery [134,200]. In addition, plasma levels of lectin pathway initiator MBL increase during the acute phase and correlate with infarct size and neurological deficits. Individuals with MBL deficiency, whether due to gene polymorphisms causing functional impairment or low expression, exhibit smaller infarcts and better recovery [200,201]. These findings highlight systemic complement dysregulation and its tissue-specific consequences in stroke.

In the acute phase, C3 promotes vascular and immune responses that contribute to secondary injury. Anaphylatoxins C3a and C5a engage their respective receptors on cerebrovascular endothelial cells, up-regulating adhesion molecules such as P-selectin and ICAM-1 and facilitating neutrophil adhesion and infiltration [63,68,133]. In middle cerebral artery occlusion (MCAO) models, genetic deletion of C3aR or C5aR attenuates these responses, reducing neutrophil influx, mitigating oxidative stress and brain edema [196,200]. C3aR blockade also preserves neuronal structure by lowering MPO-positive neutrophil accumulation [134]. Concurrently, C3a activates microglia, converting them from a surveillant to a reactive phenotype, up-regulating phagocytic receptors like CR3 and TREM2. While this enhances clearance of necrotic debris and synaptic debris, it also leads to excessive synaptic pruning [196]. C3a–microglia cross-talk also triggers a feed-forward loop of IL-1β and TNF-α production, sustaining inflammation and secondary injury. In stroke models, C3aR-deficient mice exhibit attenuated microglial activation and enhanced neuroprotection [196]. Meanwhile, aberrant deposition of C1q and C3b on functional synapses in the hippocampus and cortex promotes microglial phagocytosis and synaptic loss, impairing cognitive and motor networks [135,202].

In the subacute and recovery phases, the role of C3 becomes more nuanced. C3a signaling modulates astrocyte behavior, suppressing glial scar formation by reducing GFAP expression and promoting a neuro-supportive phenotype [133]. Stokowska *et al*. showed that delayed intranasal administration of C3a enhances Thbs4 expression, cortical rewiring, and functional recovery [133]. Simultaneously, microglia transition toward an M2 reparative phenotype under C3a influence, characterized by IL-10 and IGF-1 release and reduced phagocytic activity, facilitating axonal regeneration and remyelination [133,203]. C3a also supports neural network reorganization through promotion of neurite outgrowth, synaptogenesis, and transcallosal plasticity. Furthermore, it enhances neural progenitor cell migration and differentiation, activating intrinsic regenerative pathways in the adult brain [204].

In summary, complement C3 exerts phase-specific and cell-type-dependent effects in stroke. During the acute phase, it amplifies neuroinflammation and synaptic degeneration; in contrast, during recovery, it contributes to neurorepair through modulation of astrocytes, microglia, and progenitor cells. These findings underscore the therapeutic importance of temporally precise C3 modulation in stroke management.

### Autoimmune-associated vasculitides

C3 was also reported to play a bridging role in autoimmune-associated vasculitides (AAV) such as ANCA-associated vasculitis and systemic lupus erythematosus (SLE): it links initiating factors (autoantibodies and immune complexes) to effector mechanisms (inflammatory cell activation and tissue injury) [136,137,205]. In different diseases, C3 is activated through distinct pathways—predominantly via the alternative pathway in ANCA-associated vasculitis, characterized by prominent local synthesis and deposition, whereas in SLE the classical pathway is the major driver, leading to systemic complement consumption [206,207]. Ultimately, however, both processes converge on the deposition of complement products within the damaged vascular wall, mediating inflammatory injury [136,208].

Clinical studies have demonstrated that the level of complement C3 activation is closely linked to disease activity and organ injury in vasculitis. In ANCA-associated vasculitis, reduced plasma C3c is indicative of more severe acute kidney injury, lower estimated glomerular filtration rate, and pronounced tubulointerstitial damage, and it independently associates with interstitial arteritis [209]. In contrast, C4 levels show a weaker correlation with renal impairment, underscoring that activation of the alternative pathway plays a predominant role in ANCA-associated vasculitis pathogenesis [209]. In SLE, decreased levels of C3 and C4 have become classical indicators of disease activity, particularly reflecting activity and prognosis in lupus nephritis [210]. Histopathological studies have demonstrated that in ANCA-associated vasculitis, the localization of C3 deposition is related to disease characteristics: glomerular deposition is often associated with a worse renal function outcomes, whereas arteriolar deposition is more frequently accompanied by chronic inflammation [136,211]. In patients with SLE, the typical finding is ‘full house’ deposition, reflecting persistent activation of the classical pathway [136,212]. From a systemic inflammatory perspective, activation of the complement system closely reflects disease activity in vasculitis. In ANCA-associated vasculitis, circulating Bb levels exhibit a positive correlation with both the Birmingham Vasculitis Activity Score and the proportion of glomerular crescents, while reduced properdin levels indicate excessive consumption of the alternative pathway [205,213]. Perturbations of complement activity are further linked to coagulation abnormalities: patients with decreased C3 are more likely to develop thrombocytopenia accompanied by worsening renal function, and in SLE, uncontrolled complement activation likewise promotes thrombus formation [137,138]. Collectively, C3 activation and deposition constitute not only a hallmark pathological feature of vasculitis but also a determinant of disease activity, tissue injury, and clinical outcome. Insights from genetic and molecular studies further underscore the role of dysregulated complement control in disease susceptibility and prognosis. In ANCA-associated vasculitis, protective effects have been attributed to polymorphisms in CFB, whereas variants of factor H and its related proteins are strongly associated with severe renal injury [207,214,215]. These findings highlight that an individual’s predisposition to complement dysregulation critically shapes the risk and clinical trajectory of vasculitis.

In murine models of ANCA-associated vasculitis, factor B–deficient mice fail to develop ANCA-induced glomerulonephritis, whereas blockade of C5 or its receptor C5aR markedly attenuates glomerular necrosis and crescent formation, underscoring the indispensable role of the alternative pathway and C5a signaling in disease pathogenesis [216–218]. In SLE models, the role of complement is more complex and bidirectional: deficiency of C1q or C4 results in spontaneous lupus-like disease, indicating that early classical pathway components are protective by facilitating the clearance of apoptotic debris and preventing autoimmunity [219,220]. In contrast, once disease is established, excessive activation of C3 and C5 exacerbates glomerulonephritis and inflammatory injury, whereas genetic deficiency of C3 or C5 improves survival, highlighting the dualistic nature of complement in lupus pathophysiology [221,222].

In summary, complement C3 plays a pivotal role in autoimmune-associated vasculitides: its activation level is closely associated with disease activity, renal injury, and clinical prognosis. Genetic predisposition, local synthesis, and regulatory imbalance drive aberrant C3 activation, which synergizes with inflammasome pathways and coagulation cascades to exacerbate tissue damage. Thus, C3 emerges not only as a critical biomarker but also as a promising target for precision therapeutics.

## Therapeutic strategies targeting C3

### Mechanistic rationale for targeting C3

Complement C3 serves as the central node of the complement cascade, integrating signals from all three activation pathways and directing immune responses through its cleavage products, C3a and C3b [13,223]. In panvascular diseases, C3 is not only a driver of inflammation but also a modulator of cellular behavior and metabolic states. Its multifaceted role in orchestrating endothelial activation, immune cell recruitment, structural cell remodeling, and immunometabolic coupling makes it an attractive, yet challenging, therapeutic target [223,224]. C3a, via engagement of the G protein-coupled receptor C3aR, activates downstream effectors such as MAPKs and NF-κB in diverse cell types including endothelial cells, macrophages, cardiomyocytes, and VSMCs [223,224]. These signals drive the transcription of adhesion molecules, cytokines, and chemokines, amplifying local and systemic inflammation. C3b and iC3b, in parallel, act as opsonins facilitating clearance of apoptotic cells and modulating antigen presentation [13].

Beyond its classical immunologic functions, C3 exerts profound effects on structural cells. In VSMCs, valvular interstitial cells, and pulmonary artery fibroblasts, C3 signaling induces phenotypic switching, promoting increased motility, secretory activity, and immune evasion, traits typically associated with activated immune cells [223]. These changes reframe structural cells as active participants in inflammation and tissue remodeling rather than passive bystanders. In addition, C3 operates within the intracellular compartment through the ‘complosome’, a noncanonical complement system that regulates mitochondrial function, redox balance, and cellular metabolism [223]. In cardiomyocytes and endothelial cells, for example, intracellular C3 has been implicated in energy homeostasis and anti-apoptotic signaling. These dual extracellular and intracellular functions position C3 as a context-dependent regulator with both pathogenic and protective potential.

Given its upstream position in the complement cascade, therapeutic inhibition of C3 offers broad blockade of downstream effectors, including C3a, C5a, and MAC, while also preventing opsonization by C3b [13,225]. In the setting of cardiovascular and systemic vascular diseases, C3 is frequently activated both systemically and locally, with tissue-specific feedback loops reinforcing disease progression [225,226]. Thus, targeted inhibition of C3 holds promise for dampening inflammatory amplification and halting the progression of vascular injury.

### Current strategies for C3 inhibition

Several approaches have been developed to inhibit C3 activity with varying degrees of specificity, potency, and translational maturity:

1. Factor H mimetics: molecules such as mini-FH and TT30 mimic the activity of factor H, a natural regulator of the alternative pathway. These agents enhance degradation of C3b but are limited by their large molecular size and potential immunogenicity [227].
2. Small molecules and monoclonal antibodies: efforts to disrupt C3b-receptor interactions or stabilize convertase dissociation have faced challenges related to binding affinity, target specificity, and unintended interference with physiological pathways [227].
3. Compstatin family peptides: the most clinically advanced class of C3 inhibitors. Compstatins are cyclic peptides that bind native C3 with high affinity and prevent its proteolytic activation. These are the only C3-targeting agents that have entered human trials to date [228,229].
4. Soluble CRIg-Fc fusion proteins: These engineered molecules consist of the extracellular domain of CRIg fused to the Fc portion of IgG [230]. By binding C3b/iC3b, CRIg-Fc selectively prevents alternative pathway convertase formation. Preclinical studies have shown that CRIg-Fc can attenuate inflammation and tissue injury in models of arthritis, ischemia–reperfusion injury, and systemic lupus erythematosus [230–232].

Traditional therapies for vascular diseases may also modulate C3, though these effects are secondary to their main pharmacological actions. Statins have been shown to lower plasma C3 levels and up-regulate regulatory proteins such as DAF/CD55, thereby attenuating complement activation and endothelial injury [233–236]. Antiplatelet therapy, particularly high-dose aspirin, can similarly reduce plasma C3 and improve fibrin structure, suggesting additional vascular protection beyond platelet inhibition [237]. Moreover, RAAS inhibitors such as aliskiren and losartan decrease C3 deposition and inflammation, while C3 deficiency itself limits renin up-regulation, highlighting a bidirectional interaction [185,238,239]. Together, these findings indicate that conventional therapies may confer complementary vascular benefits by dampening C3 activation.

### Compstatins: mechanism and therapeutic advantages

First discovered in 1996 via phage display, Compstatin is a 13-residue cyclic peptide stabilized by a disulfide bridge [240]. Initial versions had moderate affinity, but subsequent structural refinements, such as amino acid substitutions (Trp, Ala, His), N-terminal acetylation, and N-methylation, led to significantly improved C3 binding [241]. Compstatins exert their effects by binding the MG4 and MG5 domains of the β-chain of native C3, thereby preventing its interaction with the C3 convertase and shielding it from cleavage into C3a and C3b [241]. This substrate-shielding mechanism not only halts upstream complement activation but also blocks the C5 convertase cascade, inhibiting downstream inflammation and cytolytic MAC formation [240].

Key pharmacological derivatives include Cp40 (also known as AMY-101) and Pegcetacoplan (APL-2). Cp40 is a third-generation compstatin with dual tryptophan substitution and N-methylation, exhibiting picomolar binding affinity and improved plasma stability [241]. Pegcetacoplan (APL-2) is a PEGylated version of Cp40 designed for extended half-life and systemic delivery, currently in advanced clinical trials [229,240]. These agents offer several pharmacokinetic and pharmacodynamic advantages, including high target selectivity, prolonged half-life, and chemical versatility. Structural studies have shown that Cp40’s saddle-shaped conformation inserts stably between the β- and α-chains of C3, anchoring via hydrophobic and hydrogen-bonding interactions and maintaining bioactivity in human plasma with a K_D in the 200 pM range [241].

### Preclinical and translational insights

While direct application of compstatins in panvascular disease is still emerging, preclinical studies in other inflammatory contexts highlight their translational potential. In lepirudin-anticoagulated whole blood models, Cp40 combined with CD14 inhibition suppressed > 70% of lipopolysaccharide-induced gene expression changes, offering superior suppression of inflammation compared with monotherapy [229]. In paroxysmal nocturnal hemoglobinuria (PNH), Cp40 blocked C3 fragment deposition and hemolysis in a dose-dependent manner, with near-complete inhibition at ~ 6 μM. In non-human primates, PEG-Cp40 demonstrated sustained C3 inhibition with a favorable half-life of up to 5 days [242]. In acute envenomation models (e.g., *Bitis arietans* venom), Cp40 treatment reduced proinflammatory mediators (IL-1β, IL-8, PGE2), suppressed NLRP3 inflammasome activation, and limited tissue damage [243]. In COVID-19-associated ARDS, AMY-101 reduced C3a/C5a and NET formation in both preclinical models and critically ill patients, alleviating lung edema and improving respiratory indices [229]. In ophthalmology, Cp40 intravitreal injection reduced C3 activation in the retina, preserving structural and functional integrity [228]. Pegcetacoplan, in trials for late-stage AMD, improved central vision and slowed disease progression, highlighting its promise for localized C3 inhibition [228].

### Safety considerations and future directions

Complement deficiencies have been associated with a spectrum of clinical complications. Deficiencies of early components in the classical pathway (including C1q, C1r, C1s, C2, and C4) are strongly linked to the development of autoimmune diseases, whereas deficiencies of C3 and its regulatory proteins are characteristically associated with recurrent, severe bacterial infections often accompanied by autoimmune manifestations [244–246]. The clinical phenotypes of these congenital defects provide valuable insights into the potential adverse consequences that may arise from long-term pharmacological complement blockade.

Despite their promise, long-term systemic C3 inhibition may impair host defense mechanisms, particularly increasing susceptibility to encapsulated bacterial infections such as *Streptococcus pneumoniae*, *Neisseria meningitidis*, and *Haemophilus influenzae*. Standard clinical protocols recommend vaccination, prophylactic antibiotics, and monitoring of hematologic and infectious parameters during therapy [225]. In addition, chronic C3 blockade may disrupt physiologic immune surveillance, apoptotic cell clearance, and tissue homeostasis [13].

To mitigate these risks, research is shifting toward localized delivery, cell-type-specific targeting, and phase-selective inhibition, especially relevant in diseases like myocardial infarction, aortic dissection, and stroke, where C3 exerts temporally distinct roles. The integration of targeted delivery systems (e.g., nanoparticles, ligand-conjugated inhibitors) and biomarker-guided timing strategies may further enhance therapeutic precision [226,240]. From a translational perspective, complement biomarker–guided patient stratification is required to delineate subpopulations that can benefit most from these interventions and to adjust therapeutic timing and duration accordingly, balancing improved clinical outcomes with reduced infectious complications [247].

In conclusion, therapeutic modulation of C3 represents a promising frontier in panvascular immunotherapy. By integrating upstream complement blockade with a nuanced understanding of tissue context, disease stage, and cellular targets, future therapies may transcend generalized suppression to achieve tailored, efficacious, and safe immune intervention.

## Conclusion and outlook

In this light, complement C3 should no longer be viewed merely as a disease-specific effector but rather as a central orchestrator of systemic vascular dysregulation. Its context-dependent activation patterns define the immunological and structural response profiles of diverse vascular territories. This perspective prompts a conceptual shift: therapeutic strategies for panvascular disease must transcend organ-specific targeting or single-pathway inhibition. Instead, future interventions should aim to modulate the C3 axis with spatial, temporal, and cell-type precision, accounting for the unique microenvironmental cues and disease stage of each vascular bed. Such an approach holds the potential to reprogram vascular inflammation and remodeling at the systems level, offering a unified yet adaptable framework for treating complex, multi-territory vascular disorders.

## Data Availability

This review is based entirely on previously published literature. No new data were generated or analyzed during the preparation of this manuscript. All data supporting the findings and conclusions are available within the cited references.

## Open Access

This article has been published open access under our Subscribe to Open programme, made possible through the support of our subscribing institutions, learn more here: https://portlandpress.com/pages/open_access_options_and_prices#conditional

## Abbreviations

C3: Complement C3
C5: Complement C5
CR3: Complement receptor 3
CRP: C-reactive protein
C3a: Complement C3a
C5a: Complement C5a
C3aR: C3a receptor
C5aR: C5a receptor
C3b: Complement C3b
Cp40: Compstatin derivative (AMY-101)
ECM: Extracellular matrix
IL-1β: Interleukin-1 beta
MAC: Membrane attack complex
MMPs: Matrix metalloproteinases
NF-κB: Nuclear factor-kappa B
NLRP3: NLR family pyrin domain containing 3
OxLDL: Oxidized low-density lipoprotein
PVAT: Perivascular adipose tissue
PVD: Peripheral vascular disease
RAS: Renin-angiotensin system
ROS: Reactive oxygen species
SHR: Spontaneously hypertensive rats
TGF-β: Transforming growth factor-beta
TNF-α: Tumor necrosis factor-alpha
VSMCs: Vascular smooth muscle cells
